# Therapeutic Editing of the *TP53* Gene: Is CRISPR/Cas9 an Option?

**DOI:** 10.3390/genes11060704

**Published:** 2020-06-25

**Authors:** Regina Mirgayazova, Raniya Khadiullina, Vitaly Chasov, Rimma Mingaleeva, Regina Miftakhova, Albert Rizvanov, Emil Bulatov

**Affiliations:** 1 Kazan Federal University, 420008 Kazan, Russia; RegMSayarova@kpfu.ru (R.M.); RaniRNazyrova@kpfu.ru (R.K.); VVChasov@kpfu.ru (V.C.); RNMingaleeva@kpfu.ru (R.M.); ReRMiftahova@kpfu.ru (R.M.); Albert.Rizvanov@kpfu.ru (A.R.); 2 Shemyakin-Ovchinnikov Institute of Bioorganic Chemistry, Russian Academy of Sciences, 117997 Moscow, Russia

**Keywords:** *TP53*, mutation, CRISPR/Cas9 gene editing, base editing, prime editing, epigenome regulation, clinical trial

## Abstract

The *TP53* gene encodes the transcription factor and oncosuppressor p53 protein that regulates a multitude of intracellular metabolic pathways involved in DNA damage repair, cell cycle arrest, apoptosis, and senescence. In many cases, alterations (e.g., mutations of the *TP53* gene) negatively affect these pathways resulting in tumor development. Recent advances in genome manipulation technologies, CRISPR/Cas9, in particular, brought us closer to therapeutic gene editing for the treatment of cancer and hereditary diseases. Genome-editing therapies for blood disorders, blindness, and cancer are currently being evaluated in clinical trials. Eventually CRISPR/Cas9 technology is expected to target *TP53* as the most mutated gene in all types of cancers. A majority of *TP53* mutations are missense which brings immense opportunities for the CRISPR/Cas9 system that has been successfully used for correcting single nucleotides in various models, both in vitro and in vivo. In this review, we highlight the recent clinical applications of CRISPR/Cas9 technology for therapeutic genome editing and discuss its perspectives for editing *TP53* and regulating transcription of p53 pathway genes.

## 1. Introduction

**Figure 1:**
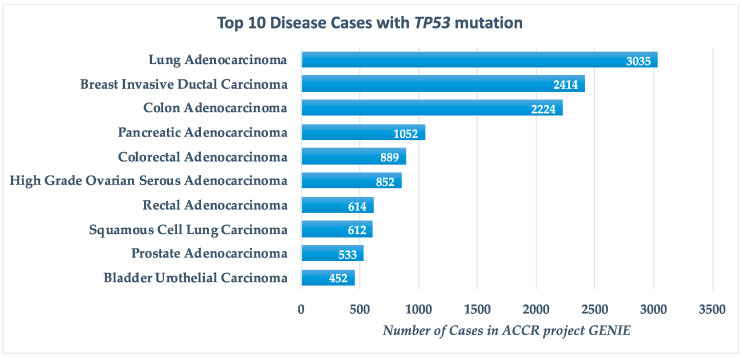
Top 10 disease cases with *TP53* mutations, according to the GENIE (Genomics Evidence Neoplasia Information Exchange) project by AACR (American Association for Cancer Research).

The *TP53* gene encodes the p53 protein, a well-known tumour suppressor involved in various regulatory pathways including cell cycle arrest, apoptosis, senescence and DNA repair. The *TP53* is the most frequently mutated gene in human cancer with mutations found in about half of all cancer cases. The Genomics Evidence Neoplasia Information Exchange (GENIE) project by the American Association for Cancer Research (AACR) reports *TP53* mutations in 32.8% of cancers, primarily lung adenocarcinoma, breast invasive ductal carcinoma and colon adenocarcinoma (Figure 1) [1].

Currently, there are over 170 ongoing clinical trials mostly for malignant solid tumours, non-small cell lung carcinoma, breast carcinoma, ovarian carcinoma and acute myeloid leukaemia in which *TP53* status serves as an inclusion eligibility criteria [1]. Among them 66 are phase I, 99 are phase II, 10 are phase III and 1 is phase IV.

**Figure 2:**
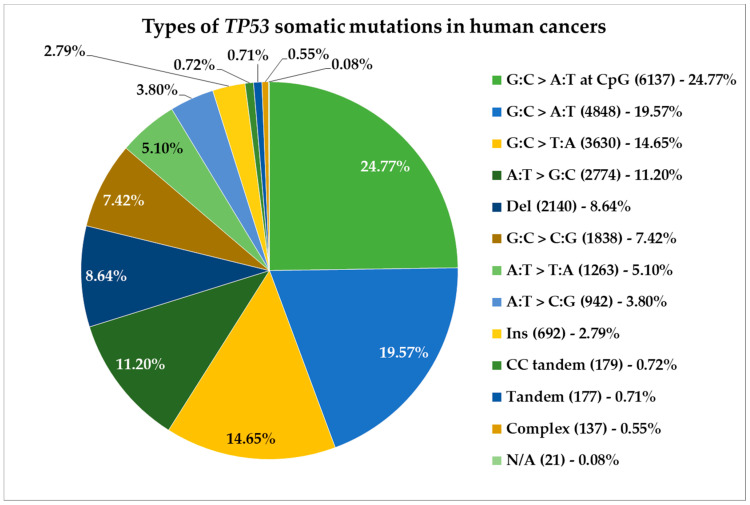
Types and distribution of nucleotide conversions in *TP53* that result in somatic mutations in human cancers, according to the IARC (International Agency for Research on Cancer) *TP53* Database.

Alterations in the *TP53* gene are commonly associated with increased risk of cancer. In most cases, only one nucleotide is mutated in the *TP53* gene leading to a single amino acid substitution in the p53 protein. The most frequent mutations are represented by G:C>A:T or G:C>T:A conversions that together amount for over half of *TP53* somatic mutations in human cancers (Figure 2) [2]. Often these mutations are frameshift and results in non-functional protein with impaired transcriptional and tumour suppressor activity.

In addition to the loss of the tumour suppressor functions, mutant p53 proteins may also promote the division of cancer cells. Shelley Berger with colleagues [3] have shown that this “gain-of-function” mutant p53 targets epigenetic regulators such as MLL1, MLL2 and MOZ, resulting in uncontrolled cell replication. This mainly affects late stage and hard to treat cancers such as pancreas, breast, brain, oesophagus, head and neck.

Interestingly, there are many cancers with WT *TP53* in which the p53 protein is inactive. Oncogenic growth of such tumours is associated with reduced p53 activity caused by overexpression of one or more epigenetic regulators that post-translationally modify p53 protein and impair its tumour-suppressor functions, including Aurora A kinase that phosphorylates p53 (S212/S312) and methyltransferases that methylate p53 such as Smyd2 (K370), Glp/G9a (K373) and PR-Set7 (K382). Inhibition of these epigenetic regulators could lead to reactivation of p53 in cancer [4].

Currently, therapeutic approaches specifically targeting the p53 protein and its negative regulators, such as MDM2, are mostly focused on small molecule modulators [5,6,7,8]; however, the emerging genome-editing technologies might soon turn into a tantamount alternative.

Gene editing as a molecular technology provides the capability to change genomic sequences in almost all types of cells. Typically, this represents DNA modifications, such as insertions, deletions and sequence substitutions, at specific locations in the genome. Several primary approaches to programmable genome editing include zinc-finger nucleases (ZFNs) [9,10], transcription activator-like effector nucleases (TALENs) [11,12] and clustered regularly interspaced short palindromic repeat (CRISPR)/Cas (CRISPR-associated) enzymes (Table 1) [13,14]. Other examples include ZFNs and TALENs, the fusion proteins consisting of DNA-binding and nuclease domains of restriction enzyme FokI, that were among the first classes of nucleases developed for precision gene modifications. However, the application of these technologies is often limited, because they are based on proteins to guide the gene editing machinery to specific DNA sequences. Therefore, targeting each new genome site requires engineering and cloning of a new protein. Currently, the CRISPR/Cas gene-editing platform based on bacterial antiviral defence mechanisms is considered to be the most prominent and elegant RNA-guided tool for efficient and specific DNA modifications. Unlike TALENs and ZFNs, DNA recognition by the CRISPR/Cas system is specified by the short-guide RNA sequence which makes this molecular tool simple and facilitates its universal use. The ultimate objective for various genetic tools is in their clinical application, since they hold a great potential for treating human diseases, especially genetic disorders and cancer.

Gene alterations often result in genetic disorders, many of which are hereditary. Although disease-associated mutations are often known, most of the current therapeutic strategies are aimed at treatment of symptoms and not correction of the mutated DNA sequence. The emerging gene-editing therapies, including CRISPR/Cas, are expected to alter human genome by correcting genetic mutations to prevent or treat the disease they cause.

CRISPR technology, originated from prokaryotic adaptive immune system against invading nucleic acids, is now widely used for biomedical research applications. CRISPR/Cas9 system, specified by the short guide RNA, has rapidly become the most popular gene-editing system due to its simplicity and flexibility. Widely used for research purposes, CRISPR/Cas9 system has long brought attention because of its immense clinical opportunities for treating diseases associated with genome alterations. Therapeutic approaches based on CRISPR/Cas technology are currently being developed for the treatment of cystic fibrosis [15], muscular dystrophy [16] and Huntington’s disease [17]. In addition, several clinical trials are ongoing for blood disorders, such as β-thalassemia and sickle cell disease, and also for inherited blindness, HIV and cancer (Table 2). Somatic mutations in *TP53* gene are the most common genetic alterations found in human cancers, such as breast and bladder cancer, head and neck squamous carcinoma, lung cancer, melanoma, ovarian cancer and many others, as well as Li-Fraumeni syndrome (LFS) that is associated with inherited mutations in *TP53* gene and hereditary cancer predisposition [18]. Here we provide a brief overview of recent advances in CRISPR/Cas technology as a promising gene-editing therapy for the treatment of cancer and hereditary diseases associated with *TP53* mutations.

## 2. Bacterial Antiviral Defence

Naturally, the CRISPR/Cas system, found in most bacteria and the majority of characterized Archaea, acts as an RNA-guided adaptive immune system against bacteriophages and plasmid transfer [19]. The CRISPR loci are short palindromic repeats (25–35 bp each) that are separated by small unique sequences called spacers (30–40 bp each). The CRISPR loci are accompanied by CRISPR-associated (Cas) genes encoding Cas proteins [20,21].

During the immunization process following an encounter with a prokaryotic cell with invading genetic elements from bacteriophages or plasmids, Cas proteins cut out a small fragment (protospacer) of this genetic element and ensure its integration into the CRISPR locus within the host chromosome as a new spacer therefore providing a genetic record of occurred invasion that enables the host to avoid such invaders in the future [22]. The next encounter with an already familiar genetic sequence triggers transcription of CRISPR RNA (crRNA) from the corresponding spacer. At 5′-end crRNA contains a spacer sequence complementary to the genetic information of the bacteriophage or plasmid (protospacer), and the 3′-end includes fragments of the repeating sequence that form hairpins. The crRNA forms a complex with the Cas effector protein; therefore, the base-pair binding of crRNA with the foreign complementary sequence triggers a sequence-specific DNA cleavage of the latter [23,24]. Notably, in most CRISPR/Cas systems, the specific target DNA recognition and cleavage requires the presence of a short-conserved sequence motif (2–5 bp), known as protospacer adjacent motif (PAM), in close proximity to the crRNA-targeted sequence of the invader [25]. To summarize, the CRISPR/Cas-based bacterial antiviral defence mechanism includes three stages: (i) adaptation, or new spacers recognition; (ii) crRNA expression and processing, or effector complex formation; (iii) interference, or foreign DNA cleavage [21,22,23,24].

## 3. Classification of CRISPR/Cas Systems

A variety of CRISPR/Cas systems are mainly associated with effector proteins that ensure the destruction of foreign DNA. According to a recent report, CRISPR/Cas systems are divided into two major classes based on the differences in effector (endonuclease) module components and mechanisms of action [21]. In class 1, several subunits form a large multi-Cas effector complex, whereas in class 2, it consists only of a single multi-domain protein. These classes are further divided into three types each, as described below (Table 1).

Class 1 and 2 differ in the mechanism of pre-crRNA processing, among other aspects. In class 1, the nuclease responsible for processing is Cas6, whereas in class 2 it depends on the type and may vary. In type II processing of pre-crRNA is catalysed by an external bacterial enzyme, RNase III, in types V and VI, the processing is mediated by nuclease domain of the large effector protein.

Other differences between class 1 and class 2 appear to be involved in the interference stage. In class 1, the nuclease responsible for target cleavage is Cas3 (type I) or a subunit of the processing complex itself (type III). In class 2, cleavage is performed by the nuclease domain of the effector protein [13,14,26].

Most effectors target DNA, only one—exclusively RNA—rarely both DNA and RNA. The widely known CRISPR/Cas9 system is an example of type II (class 1) Cas9 effector protein that cleaves dsDNA [14,21,27].

## 4. Components and Mechanism of CRISPR/Cas9 System

The CRISPR/Cas9-targeted genome-editing system consists of two main components: a short-guide RNA (sgRNA or gRNA) and Cas9 endonuclease. The most studied and commonly used *S. pyogenes* Cas9 is a large (1368 aa) multidomain endonuclease that cleaves dsDNA 3 bp upstream of PAM and possesses two distinct nuclease domains: HNH-like nuclease responsible for cleaving the DNA strand complementary to sgRNA (target strand) and RuvC-like nuclease that cleaves the opposite DNA strand [27].

Importantly, despite containing PAM-interacting sites, Cas9 remains inactive and unable to bind the target DNA sequence without the presence of sgRNA [28]. Target DNA recognition requires complementarity between 20 bp sgRNA spacer and protospacer of the target DNA site that also has an adjacent PAM sequence [13]. The target DNA recognition process begins with probing for a suitable PAM sequence by Cas9, and when it is found, the DNA is interrogated for complementarity to sgRNA. After Cas9 has found the target site, it triggers local DNA melting next to PAM, followed by binding of sgRNA to the target site, formation of RNA–DNA duplex and DNA strand displacement (formation of R-loop) [28,29]. Recognition of PAM and RNA–DNA hybridization activates Cas9 endonuclease for dsDNA cleavage resulting in double-strand breaks (DSBs) at the target locus [24].

## 5. DSB Repair Mechanisms

The DNA DSBs are generated as a result of the phospho-sugar backbone cleavage of both DNA strands either at the same positions or in relative proximity, which leads to physical unwinding of DNA double helix. Unlike DNA single-strand breaks (SSBs), DSBs cause greater cell damage because they are more difficult to repair without the loss of genome integrity, since there is no complementary chain with genetic information available as a repair template [30].

Upon cleavage by Cas9, there are two possible mechanisms for subsequent DNA damage repair of the target locus: the efficient but error-prone non-homologous end joining (NHEJ) and the less efficient but high-fidelity homology-directed repair (HDR) [31]. The primary difference between these DNA repair mechanisms is the utilization of a template sequence to join the DSB ends, as in case of HDR [32].

In vertebrate cells NHEJ is a common DNA repair mechanism that occurs throughout the cell cycle. This mechanism does not require the homology sequence template, since through the NHEJ process a group of proteins bind to DSB blunt ends resulting in re-ligation of DNA break [33,34]. In most cases, NHEJ results in insertions or deletions (indel) at the DSB site.

In contrast to NHEJ, the HDR mechanism is generally less efficient, because it requires cell division, presence of homology sequence template prior ligation and can vary significantly depending on the cell type and state, genomic locus, and repair template. Both repair mechanisms are differentially regulated according to the phase of cell cycle, e.g., HDR is limited to S/G2, while NHEJ is functional throughout the entire cell cycle [35].

Microhomology-mediated end joining (MMEJ) is an alternative to NHEJ repair mechanism that relies on recombination of exposed microhomology sequences (5–25 bp) flanking the broken junction to repair DSBs [36]. However, MMEJ is often associated with unintended errors, such as deletions, insertions, mutations and chromosome translocations.

## 6. Applications of CRISPR/Cas9 System

### 6.1. CRISPR/Cas9-Mediated DSB Repair Mechanisms

**Figure 3:**
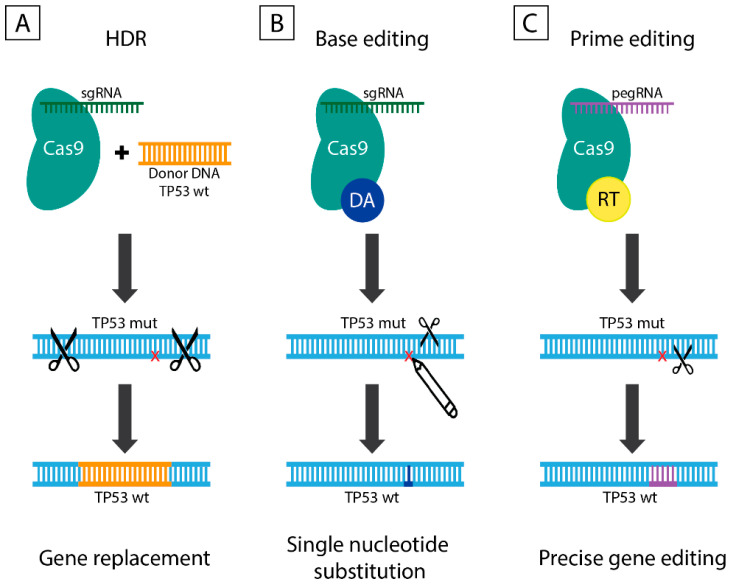
The diverse approaches for *TP53* gene editing: DSB-mediated gene replacement by a normal copy, precise *TP53* editing by base editors and prime editors that do not require DSBs. (**A**) HDR is a DSB-mediated DNA repair mechanism that requires exogenously introduced homology sequence template prior ligation. HDR is active in S/G2 phase of the cell cycle and allows target sequence modifications ranging from a single nucleotide substitution to insertion of large nucleotide sequences into a target locus. (**B**) Base editors consist of a nickase Cas9 (nCas9 with D10A mutation) fused to either cytidine deaminase or adenosine deaminase and mediate C to T or A to G conversion in genomic DNA, respectively. (**C**) Prime editors exemplify a so-called “search-and-replace” technology, whereby new genetic information is directly introduced by means of nCas9-RT fusion protein guided by pegRNA that both specifies the target DNA sequence and encodes the desired edits. DA—deaminase; DSB—double-strand break; RT—reverse transcriptase.

Nucleotide sequence modifications by the CRISPR/Cas9 system can be achieved by NHEJ, HDR and MMEJ which are described here and below as CRISPR/Cas9-mediated DSB repair mechanisms. The NHEJ is primarily used to generate gene knockouts, since indels cause frameshift mutations resulting in premature stop codons within the open reading frame of the gene of interest. The expected result of NHEJ repair is a loss-of-function mutation of the target gene [37]. Unlike NHEJ, the application range of HDR is substantially wider, since besides knockouts it also allows producing precise genetic modifications by means of an exogenously introduced repair template (Figure 3A). The HDR can induce alterations varying from substitution of a single nucleotide to insertion of a desired sequence into the target locus. Depending on the application, the repair template can represent ssDNA oligonucleotide or dsDNA with homology arms flanking the insertion site [38]. The efficiency of CRISPR/Cas9-mediated DSB repair mechanisms remains relatively low and is usually associated with frequent side effects due to the non-specific DSBs beyond the target site. Despite all the shortcomings, the simplicity and flexibility of CRISPR/Cas9-mediated DSB repair mechanisms still allows researchers to enhance efficiency and improve accuracy by varying sgRNA and Cas endonuclease.

### 6.2. Base Editing

The gene-editing approaches described below are based on the introduction of DSBs at the target DNA site as a starting point of the mechanism. Moreover, precise gene correction using NHEJ is extremely difficult due to the fact of its stochastic nature. Similarly, the efficiency of HDR for point gene corrections remains very low largely because of DSBs generated at the target locus.

In 2016, the laboratory of David Liu from Harvard University proposed an alternative tool for precise gene correction, named base editing, which enables programmable conversion of one nucleotide to another without the need for DSB or homology sequence template [39]. Base editors (BEs) are fusion proteins of cytidine/adenosine deaminase with catalytically inactive Cas9 (dCas9 with D10A and H840A mutation) or nickase Cas9 (nCas9 with D10A mutation) (Figure 3B). The dCas9 is a non-functional nuclease that still retains the ability to bind DNA in an sgRNA-mediated manner. BEs are more efficient (up to 15–75% in human cells) in comparison to classical CRISPR/Cas9 tools and demonstrate high fidelity, low indel rates (<0.1%).

There are two classes of BEs–cytosine base editors (CBEs) [39] that convert C to T (or G to A on the opposite strand) and adenine base editors (ABEs) [40] that mediate conversion of A to G (or T to C) in genomic DNA. The CBEs employ dCas9 fused to rat cytidine deaminase APOBEC1 that mediates C to U conversion within a narrow activity window (range of nucleotides amenable to deamination). These enzymes can also include uracil-DNA glycosylase (UDG) to inhibit reversion of U:G back to C:G that commonly occurs in cells. In this way, dCas9–UDG fusion protein mediates U:G to T:A conversion in the course of DNA replication [39].

Certain types of nucleotide transformations can be more difficult to achieve than the others. Surprisingly, only RNA adenine deaminases exist in nature and they do not process DNA as a substrate. The same team of scientists led by David Liu [40] obtained DNA adenine deaminases through directed evolution of *E. coli* TadA, a tRNA adenosine deaminase, and developed dCas9-TadA fusion ABEs that produce A to G substitution in genomic DNA. The most widely used ABE7.10 is a fusion of nCas9 and heterodimeric TadA (wt/mutant complex). The ABEs catalyse conversion of A to I, which is then recognized as G by DNA polymerase ultimately resulting in A to G substitution.

Each of the described base editing tools offers different gene correcting options according to the type of deaminase, Cas9 nuclease and sgRNA employed. Typically, BE3 and ABE7.10 demonstrate the highest efficiency at protospacer positions 4–8 and 4–7 (considering PAM at positions 21–23), respectively.

### 6.3. Prime Editing

Although considered much safer and more precise than traditional CRISPR/Cas9 technology, BEs remain limited in their applications. The BEs can be efficiently used to correct four transition mutations (C to T, G to A, A to G, and T to C) without introducing DSBs and the need for donor DNA template; however, they are unable to edit eight transversion mutations (C to A, C to G, G to C, G to T, A to C, A to T, T to A, and T to G). In addition, BEs cannot be used to perform insertions and deletions, which along with transversions would account for the majority of gene-correcting approaches to target pathogenic alleles.

In late 2019, the laboratory of David Liu [41] offered the latest alternative to DSB-mediated modifications—prime editing technology based on “search-and-replace” strategy (Figure 3C). Prime editors (PEs) allow precise and efficient base-to-base conversion for all 12 possible variations, including insertions, deletions, and combinations thereof without the requirement for DSB or DNA template. This gene editing system consists of three main components: (i) prime editing extended guide RNA, or pegRNA, that functions as both sgRNA and donor template for the desired alteration; (ii) fusion protein consisting of Cas9 nickase and optimized M-MLV reverse transcriptase (RT); (iii) sgRNA that mediates cleavage of non-edited DNA strand by Cas9 nickase. Three generations of PEs include PE1 that utilizes RT fused to nCas9 and pegRNA, PE2 based on optimized RT with increased efficiency, and PE3 that nicks the non-edited strand to induce its replacement and further enhance the efficiency.

Briefly, nCas9-RT fusion guided by pegRNA nicks a single DNA strand at a precise position, then RT polymerizes the nicked strand using pegRNA as a template. After that the second sgRNA targets Cas9 nickase for the non-edited strand to form SSB, thereby inducing its repair and introduction of the desired modification in both DNA strands [41].

The PEs provide higher efficiency and fewer off-target edits than CRISPR/Cas9-mediated DSB repair mechanisms. Moreover, in contrast to BE- or HDR-based approaches, PEs do not require PAM sequence in close proximity to the target site. Thus, they possess a clear advantage over the existing CRISPR/Cas9 technologies by allowing gene editing within up to 30 bp away from the PAM sequence. However, PEs are only effective for small insertions/deletions (<100 bp). Ironically, despite their low efficiency and considerable inaccuracy currently only HDR-based techniques currently permit large [42].

## 7. CRISPR/Cas9 System for Gene Regulation

The highly adaptive CRISPR/Cas9 technology presents a wide range of potential applications because of the ability of modified Cas enzymes to bind DNA without cleaving the double-stranded helix. In addition to genome editing, CRISPR/Cas9 can be used for regulation of transient and stable gene expression in a sequence-specific, non-mutagenic manner. Transcription process can be altered via CRISPR/Cas9-mediated mechanism not by modifying the genomic sequences, but rather through the recruitment of effector proteins capable of transcriptional repression and activation, epigenome editing.

### 7.1. Transcriptional Activation and Repression

**Figure 4:**
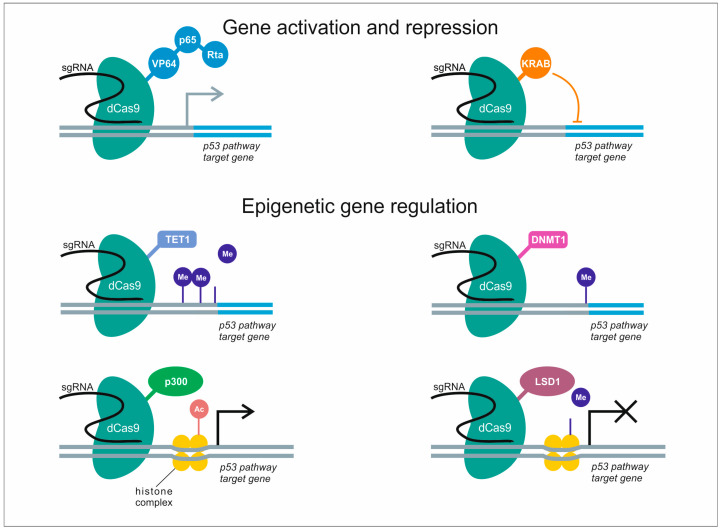
Potential applications of CRISPR/dCas9 system for regulation of p53 pathway genes. dCas9-mediated regulation of gene expression is guided by sgRNA, whereby dCas9 can be fused to a variety of transcriptional modulators to activate (VP64, p65, Rta) or repress (KRAB) target gene transcription. In addition, dCas9 fused to histone-modifying enzymes can modulate (TET1, DNMT1), activate (p300) or inhibit (LSD1) p53 pathway gene expression by epigenetic regulation.

The regulation of gene transcription functions by CRISPR/Cas9 tools is based on dCas9 that lacks the nuclease activity but can still be guided by sgRNA to bind the target DNA sequence. Following the same principle as for BEs/PEs, dCas9 could be fused with a variety of other enzymes or transcription factors to mediate site-specific regulation of gene expression. This includes modulation of downstream gene expression by means of transcriptional activation (CRISPR activation, or CRISPRa), e.g., VP64, P65, Rta, or transcriptional repression (CRISPR interference, or CRISPRi), for example, KRAB (Figure 4). The regulatory proteins can be either fused to dCas9 or to RNA-binding proteins (RBPs) recruited to the target site through interaction with a hybrid RNA scaffold coupling sgRNA and RNA aptamer (e.g., MS2, PP7) [43]. Transcriptional repression can be achieved by dCas9-KRAB fusion protein that recruits chromatin-modifying complexes to enhance CRISPRi silencing of gene expression [44].

### 7.2. Epigenetic Regulation

Flexible and adaptive CRISPR/Cas9 system can be employed to generate tools for epigenome engineering by fusing inactive Cas9 to epigenetic regulators, e.g., p300, LSD1, MQ1, DNMT, and TET1. These include target-specific DNA demethylation by TET1 [45] and methylation by DNMT [46], especially in close proximity to CpG islands (CGIs) of the promoter regions. In addition, histone acetylation inducers such as p300 and LSD1 weaken the interaction between the histone and the DNA to facilitate access for transcription factor proteins to specific genomic locations (Figure 4). Histone methylation may have opposite effects depending on the amino acid residue, e.g., K4 methylation of H3 upregulates gene expression, while K27 methylation is inhibitory [47,48]. Overall, these approaches can be used not only to regulate gene expression, but also to elucidate the causal relationship between various epigenetic marks and their respective phenotypic outcomes.

## 8. Clinical Trials of CRISPR/Cas9 Therapeutic Genome Editing

Oncogenic or disease-causing mutations represent highly promising targets for gene-editing therapies. However, one of the main barriers on the path for wider clinical application of CRISPR/Cas9 technologies is the intracellular delivery. Currently, there are three main strategies for delivering components of the CRISPR/Cas9 system into cells and tissues that include: (i) viral vectors for DNA encoding the enzyme and sgRNA; (ii) lipid nanoparticles for mRNA encoding the enzyme and sgRNA; (iii) Cas9 with sgRNA as a preformed ribonucleoprotein complex. Among these the viral gene delivery strategy is the closest to clinical application, since it has been used in classical gene therapy for decades [49].

The first CRISPR/Cas9 gene-editing therapies tested in clinical trials (NCT03745287, NCT03655678, NCT03399448) were developed for the treatment of haematological diseases because in this case manipulations with blood cells are safely performed ex vivo and then gene-modified cells are infused back to the patient.

Several clinical trials are currently in progress to apply CRISPR/Cas9 for the treatment of blood disorders and other diseases (Table 2). A notable case is CTX001, an investigational ex vivo CRISPR/Cas9 gene-editing therapy for modification of *BCL11A* gene in autologous CD34+ human hematopoietic stem and progenitor cells (hHSPCs). The CTX001 is currently being tested for the treatment of sickle cell disease (NCT03745287) and β-thalassemia (NCT03655678), and the latest clinical advances reported in mid-June 2020 demonstrated promising results: two patients with β-thalassemia and one with sickle cell disease no longer required blood transfusions. Another clinical trial (NCT03728322) evaluates transplantation of induced hematopoietic stem cells (iHSCs) with CRISPR/Cas9-edited *HBB* gene for the treatment of β-thalassemia.

Genetic modification of T and B cells to treat various types of cancers or HIV is another promising avenue for clinical application of CRISPR/Cas9 technology (Table 2). Examples of other CRISPR/Cas9-mediated clinical therapies include allogeneic anti-CD19 CAR-T cells with disrupted *TCR* and *B2M* genes (NCT03166878), PD-1 knockout T cells (NCT02867345, NCT02867332, NCT03044743), CRISPR/Cas9-edited cell therapy CTX110 (NCT04035434), CD34+ cells with modified *CCR5* gene for HIV treatment (NCT03164135).

In February 2020, Carl June with colleagues [50] reported a first-in-human clinical trial (NCT03399448) proposed to evaluate HLA-A*0201 restricted NY-ESO-1 redirected T cells with CRISPR/Cas9-edited endogenous T cell receptor (TCR) and PD-1 for treatment of relapsed refractory multiple myeloma and other cancers. In this study gene-modified T cells persisted in patients for up to 9 months, indicating low immunogenicity and demonstrating the feasibility of CRISPR/Cas9 gene editing for cancer immunotherapy.

Another prominent example includes AGN-151587 (also known as EDIT-101), the world’s first in vivo CRISPR/Cas9-based investigational therapy for the treatment of Leber congenital amaurosis 10 (LCA10) administered via subretinal injection (NCT03872479). The LCA10 genetic disease is a common form of inherited childhood blindness caused by c.2991+1655A>G mutation in *CEP290* gene, that results in the retinal degeneration. The gene-editing therapy AGN-151587 is currently being clinically evaluated to eliminate this pathogenic *CEP290* mutation (Table 2). In March 2020 AGN-151587 treatment of the first patient was announced which marked a great progress for in vivo human gene therapy. The far-reaching consequences of this early achievement will expand our arsenal of therapies for the treatment of solid tissue-associated genetic disorders whereby cells cannot be isolated for ex vivo manipulations and subsequent re-infusion like in haematological diseases.

## 9. CRISPR/Cas9 System and *TP53* Pathway Genes

Transcription factor p53 functions as tumour suppressor and is considered as one of the most promising molecular targets for cancer therapy as it regulates a plethora of intracellular metabolic pathways, e.g., DNA damage repair, apoptosis, senescence.

### 9.1. p53 Pathway Affects the Efficiency of CRISPR/Cas9 Gene Editing

The contribution of p53 pathway to the functioning of CRISPR/Cas9 system was revealed recently, although, in as early as 1994 Di Leonardo et al. [51] stated that a single DSB is enough to induce p53-dependent cell cycle arrest in normal human fibroblasts. One of the reasons for low genome-editing efficiency of the classical CRISPR/Cas9 system is the functioning of cellular DNA repair mechanisms [52,53]. In some cells DSBs of the genomic DNA can lead to cell cycle arrest or cell death via p53 pathway that induces DNA damage response (DDR) and activates expression of downstream effector proteins, e.g., cell cycle inhibitor p21. Apparently, cells with CRISPR/Cas9-mediated DSBs, which is often an integral initial step of the gene-editing mechanism, can also be affected by DNA repair machinery with resulting loss of successfully edited cells.

A recent report by Sinha et al. [54] addressed the question of whether CRISPR/Cas9 editing could lead to oncogenic mutations in other genes. Authors analysed genome-wide CRISPR and RNAi screens to reveal the mutation selection potential of CRISPR knockouts across the whole exome. They found that CRISPR gene editing induces p53-dependent damage and can select for *KRAS* and *VHL* mutants, at a level comparable to that of *TP53*. Finally, they performed a pooled and arrayed CRISPR screens to evaluate the competition between CRISPR-edited isogenic *TP53* WT and mutant cell lines, testifying the findings by showing enhanced growth of *TP53* mutants compared to WT in a co-culture. The findings suggest that a cautionary monitoring of these three driver genes status is required during CRISPR/Cas9 editing for cell-based therapy, especially when applied to genes with a high risk of inducing mutation selection.

Bowden et al. performed parallel screens in wild-type and *TP53* knockout human retinal pigment epithelial cells to assess the impact of p53 status on the gene-editing efficiency of CRISPR/Cas9 system [55]. To interrogate the p53-mediated responses to CRISPR/Cas9-associated DSBs authors designed a dual guide RNA library targeting 852 DDR-related genes. They demonstrated that a p53-mediated DDR negatively affects the sensitivity of CRISPR/Cas9 screens used for identification of depleted genes. Nevertheless, authors ultimately suggest that the optimization of screen design would still allow successful CRISPR/Cas9 screens to be conducted in both p53 wild-type and p53-deficient cells. Hence, deeper understanding of how cellular p53 status affects the efficiency of CRISPR/Cas9 machinery will certainly facilitate the development of future gene-editing therapies.

Interestingly, Cas9 was reported to activate the p53 pathway and select for p53-inactivating mutations [56]. In this study, gene expression profiling of human cancer cell lines and their Cas9-expressing derivatives identified upregulation of the p53 pathway upon introduction of Cas9, particularly in *TP53* WT cell lines. Generally, Cas9 was less active in *TP53*-WT than in *TP53*-mutant cell lines and in some cases introduction of Cas9 resulted in the formation and expansion of p53-inactivating mutations. These findings complement previous report suggesting that CRISPR-induced p53 pathway activation could be overcome by transient p53 silencing [57] and could have major consequences for therapeutic applications of CRISPR/Cas9 genome editing.

### 9.2. Perspectives of Applying CRISPR/Cas9 System for TP53 Editing and Regulation of p53-Pathway Genes

The *TP53* gene that encodes p53 protein is mutated in approximately half of all human cancers, which makes it a highly desirable target for gene-editing tools, e.g., to reverse pathogenic mutations back to the wild-type (WT) state [58].

Chira et al. [59] proposed a theoretical concept for CRISPR/Cas9-based delivery system of functional *TP53* gene that yet has to be validated in practice. According to this, dysfunctional mutant *TP53* gene could be replaced by a fully functional wild-type copy through homologous recombination leading to normal expression of oncosuppressor p53 protein. Theoretically, this might be feasible because the capability of CRISPR/Cas9 system indeed allows making such large insertions [60,61].

Zhan et al. [62] reported CRISPR/Cas9-based genetic tool that specifically eliminated p53-deficient cells. Authors constructed a p53 genetic sensor that specifically detected the cellular expression of WT p53. The p53 sensor was combined with diphtheria toxin (DT) using the CRISPR-Cas9 system to specifically kill p53-deficient tumour cells by getting activated in presence of WT p53, therefore protecting normal cells from DT. Potentially, such sensor-associated genetic tools could be used as anti-tumour agents targeting cells that do not express WT p53.

A recent computational study reports a statistical framework called “Computational CRISPR Strategy” (CCS) for fine mapping of DNA fragments as potential p53-dependent critical enhancer regions (CERs) involved in regulation of gene expression [63]. For that they constructed a p53 CRISPR enhancer dataset for training and testing a computational CRISPR model. Authors speculate that such an approach might be useful for CRISPR screen experiments aimed to identify a small number of target genomic regions among massive genome-wide regions. Analysis indicated that top-ranked 7-mers were mapped onto informative transcription factor motifs, including *POU5F1* encoding for Oct4 that maintains the pluripotency of human embryonic stem cells by inactivating p53 through Sirt1-mediated deacetylation.

The CRISPR/Cas9 genome editing, including base editing, prime editing and upcoming technologies, offers encouraging opportunities for future clinical applications. The BEs and PEs, which allow precision correction of a target genomic locus without deleting the gene, could potentially be used to edit *TP53* missense mutations as a prospective anticancer gene therapy.

Given the high mutation rate of *TP53* gene across all cancers it is crucial to reveal how this might affect the potential therapeutic aspects of various gene-editing technologies, primarily CRISPR/Cas9. The p53 functions are delicately balanced to facilitate CRISPR/Cas9 editing while protecting the genome against the tumorigenic DNA damage. Therefore, it is often critical to understand the actual cellular p53 status and spontaneous *TP53* mutation rate for successful CRISPR/Cas9 editing. As a confirmation of this, human embryonic stem cells can spontaneously acquire cancer-associated dominant-negative *TP53* mutations under normal cell culture conditions [64]. The expected boost in the number studies revealing the capabilities of CRISPR/Cas9 for editing of *TP53* gene with respect to the treatment of hereditary diseases might urge the need for clinical validation of germline genome editing on human embryos, a topic of hot debate.

As a precaution, Alanis-Lobato et al. [65] from the Francis Crick Institute have just reported a computational pipeline for single-cell genomics and transcriptomics of OCT4 (*POU5F1*) CRISPR/Cas9-targeted and Cas9-only control human preimplantation embryos that allowed them to evaluate hard-to-detect on-target mutations. They observed loss-of-heterozygosity, segmental loss and gain of chromosomes, as well as unintended genome editing in analysed human embryo cells. The results emphasize the need for further studies to assess the safety of genome editing techniques in human embryos with a long-term objective to prevent potential pathologic consequences in clinical applications.

## 10. Conclusions

With the introduction of CRISPR/Cas9 technology genome editing has reached a new level of accessibility for a wider scientific community. The technology turned out to be simple, flexible, and cost-effective; it continues evolving to improve accuracy and reduce off-target effects. Before our eyes the natural phenomenon has been successfully adapted by the scientists to transform into a useful biomedical toolkit for diverse research needs and one ultimate goal—to cure human diseases.

Successful clinical application of CRISPR/Cas9 for genetic disorders, such as sickle cell disease and β-thalassemia, validated the therapeutic strategy for genome editing and paved the way for targeting cancer and hereditary diseases associated with mutated or impaired *TP53* gene. Classic cancer predisposition disorders like Li-Fraumeni syndrome are commonly caused by germline mutations of *TP53,* the most frequently mutated gene in human cancer. Correcting these point mutations or regulating the *TP53* pathway by epigenetic effectors that can activate or repress transcription of the target genes appears to be a promising therapeutic approach for genomic intervention. Base editors and prime editors for precision correction of single nucleotides demonstrated highly encouraging results in vitro and are now being adapted for in vivo therapeutic administration. In addition, dCas9 fused to transcriptional modulators or histone-modifying enzymes could be used for regulation of *TP53* pathway genes, which expands the scope of potential applications beyond correction of point mutations.

Until recently most of the CRISPR/Cas9 clinical therapies were limited to ex vivo manipulations with blood cells. However, the latest in vivo achievements, e.g., with LCA10 eye disorder, bring us closer to direct genome-editing applications inside the human body. Overall, accelerated advancement of CRISPR/Cas9 technologies for *TP53*-associated pathologies will enhance precision, enable improved correction of point mutations and regulation of epigenome, provide better delivery, reduce side effects and facilitate wider clinical application.

## Figures and Tables

**Table 1:** The classification of CRISPR/Cas systems.

		Effector Module	Pre-crRNA Processing	Interference Stage (Target Cleavage)
Class 1	Type I	Multi-subunit effector complex	Cas6	Cas3
	Type III			Subunit of the processing complex
	Type IV			Uncharacterized
Class 2	Type II	Single multi-domain protein	RNase III	Nuclease domain of the effector protein
	Type V		Nuclease domain of the effector protein	
	Type VI			

**Table 2:** Clinical trials of gene-editing therapies based on CRISPR/Cas9 technology.

Disease	Clinical Trial Number	Therapy	Study Start Date	Comment
Sickle cell disease	NCT03745287	CTX001 (autologous CD34+ hHSPCs with CRISPR/Cas9-modified *BCL11A* gene)	November 2018	Active
β-Thalassemia	NCT03655678	CTX001 (autologous CD34+ hHSPCs with CRISPR/Cas9-modified *BCL11A* gene)	September 2018	Active
β-Thalassemia	NCT03728322	iHSCs with CRISPR/Cas9-modified *HBB* gene	January 2019	Active
B cell leukaemia, B cell lymphoma	NCT03166878	UCART019 (universal CRISPR/Cas9-edited anti-CD19 CAR-T cells)	June 2017	Active
Prostate cancer	NCT02867345	PD-1 knockout T cells	November 2016	Withdrawn
Renal cell carcinoma	NCT02867332	PD-1 knockout T cells	November 2016	Withdrawn
Epstein–Barr virus (EBV) associated malignancies	NCT03044743	PD-1 knockout T cells	April 2017	Active
B cell malignancies	NCT04035434	CTX110 (CRISPR/Cas9-edited T cells)	July 2019	Active
HIV-1	NCT03164135	CRISPR/Cas9 *CCR5* gene modified CD34+ hematopoietic stem/progenitor cells	May 2017	Active
Multiple myeloma, melanoma, synovial sarcoma, myxoid/round cell liposarcoma	NCT03399448	NY-ESO-1 redirected autologous T cells with CRISPR/Cas9-edited endogenous TCR and PD-1	September 2018	Terminated
Leber congenital amaurosis 10	NCT03872479	AGN-151587 (correction of c.2991+1655A>G mutation in *CEP290* gene)	September 2019	Not recruiting, active

CRISPR/Cas9—clustered regularly interspaced short palindromic repeats/CRISPR-associated.

## Abbreviations

AACR—American Association for Cancer Research; ABE—adenine base editor; BE—base editor; CAR-T—chimeric antigen receptor T cell; Cas—CRISPR-associated; CBE—cytosine base editor; CCS—computational CRISPR strategy; CER—critical enhancer region; CGI—CpG island; CRISPR/Cas—clustered regularly interspaced short palindromic repeats/CRISPR-associated; crRNA—crispr RNA; dCas9—dead Cas9; DA—deaminase; DDR—DNA damage response; DNMT1—DNA (cytosine-5)-methyltransferase 1; DSB—double strand break; DT—diphtheria toxin; EBV —Epstein–Barr virus; HDR—homology directed repair; GENIE—genomics evidence neoplasia information exchange; hHSPC—human hematopoietic stem and progenitor cells; Indel—insertion/deletion; KRAB—Krüppel associated box; LCA10—Leber congenital amaurosis 10; LFS—Li-Fraumeni syndrome; LSD1—Lysine-specific histone demethylase 1; nCas9—Cas9 nickase; NHEJ—non-homologous end joining; MMEJ—microhomology-mediated end joining; PAM—protospacer adjacent motif; PE—prime editor; pegRNA—prime editing guide RNA; RBP—RNA-binding protein; RT—reverse transcriptase; sgRNA (gRNA)—short guide RNA; TALEN—transcription activator-like effector nuclease; TCR—T cell receptor; TET1—translocation methylcytosine dioxygenase 1; UDG—uracil-DNA glycosylase; WT—wild-type; ZFN—zing-finger nuclease.
